# Evaluation of Large Language Models in the Diagnosis, Urgency Triage, and Initial Management of Ophthalmic Emergencies

**DOI:** 10.7759/cureus.101433

**Published:** 2026-01-13

**Authors:** Surina Mittal, Yakshi Aggarwal

**Affiliations:** 1 Ophthalmology, Oxford University Hospitals NHS Foundation Trust, Oxford, GBR; 2 Medicine, Oxford University Hospitals NHS Foundation Trust, Oxford, GBR

**Keywords:** artificial intelligence (ai), healthcare technology, large language model (llm), ocular emergency, ophthalmology

## Abstract

Introduction

Artificial intelligence (AI) technologies are progressing rapidly and becoming an integral part of how healthcare professionals obtain medical knowledge. Large language models (LLMs) now enable clinicians to have direct access to medical guidance and support in clinical reasoning. In ophthalmology, where prompt identification of sight-threatening symptoms is essential, these tools can offer diagnostic support, urgency triaging, and initial management guidance, thus potentially reducing delays in care and improving referrals. Limited evidence exists regarding their accuracy, reliability, and safety in eye emergencies. This study aims to compare the diagnostic accuracy, urgency triage, and initial management advice generated by the three leading LLMs, to evaluate their prospective role in the early assessment and management of acute eye presentations.

Methods

This cross-sectional study compared the performance of three LLMs, including ChatGPT-5 (2025, OpenAI, San Francisco, CA, USA), Google Gemini 2.5 Pro (2025, Google DeepMind, London, UK), and Claude Opus 4.1 (2025, Anthropic, San Francisco, CA, USA), using a set of 40 standardised ophthalmic emergency vignettes across five key subspecialties within ophthalmology. Each vignette was entered into each LLM, and responses were evaluated for diagnostic accuracy (2 points), urgency recognition (2 points), initial management advice (3 points), and identification of red flag symptoms (1 point). Each vignette case had a minimum possible score of 0 and a maximum possible score of 8. Scores were compared across the three models, and statistical significance was assessed using the Wilcoxon signed-rank and Friedman tests.

Results

In this analysis, 40 clinical vignettes were each evaluated across three LLMs: ChatGPT-5, Gemini 2.5 Pro, and Claude Opus 4.1, producing 120 responses in total. Overall scores were similar across ChatGPT (6.88 ± 1.16), Gemini (7.03 ± 1.21), and Claude (6.93 ± 1.19), with no significant differences identified on statistical analysis. Additional comparison across diagnostic scores, urgency triage, red flag recognition, and management scores yielded no significant differences between any of the LLMs. Further subgroup analysis comparing subspecialties similarly yielded no significant differences across all LLMs.

Conclusion

This study demonstrates that ChatGPT-5, Google Gemini 2.5 Pro, and Claude Opus 4.1 show consistent performances in diagnosing, triaging, and providing management advice for ophthalmic emergencies from text-based clinical vignettes. All models achieved diagnostic accuracies above 80% and provided management advice in line with recognised ophthalmology guidelines, with no statistically significant differences between their overall performances. These findings showcase the potential of LLMs as support tools for ophthalmic clinical advice, particularly for non-specialists, where guidance is valuable. However, their diagnostic errors and consequent suboptimal management advice emphasise the need for ongoing development and human supervision to ensure safety before widespread clinical application.

## Introduction

Artificial intelligence (AI) is a rapidly developing field, involving computer-based systems emulating human cognitive processes such as learning, reasoning, and problem-solving [1]. The ability of AI to recognise patterns from comprehensive datasets and generate informed, evidence-based responses is a key rationale for its rapid introduction into the medical field. Modern medical practices are embracing AI, with common uses including the detection of disease from radiographic imaging, clinical decision making, and prediction of epidemiological disease patterns [2]. AI systems are commended for their adaptability and continuous learning as more data is entered, allowing them to be used in a field such as medicine, in which continuous advances and improvements are made [3].

Over the last decade, progress in AI technology has allowed for the development of more sophisticated systems, including the launch of large language models (LLMs) [4]. LLMs can interpret and generate human-like text to provide accessible responses in any style and context requested by the user. The release of the ChatGPT LLM in 2022 sparked global attention to the potential of AI, with public interest in AI systems surging, and many subsequent competitor systems, such as Google Gemini, Claude, and Microsoft Copilot, being released in quick succession [4,5]. AI models are being continuously developed to increase their ability to receive multimodal inputs and to improve the overall quality of their outputs. ChatGPT-5 (2025, OpenAI, San Francisco, CA, USA), Google Gemini 2.5 Pro (2025, Google DeepMind, London, UK), and Claude Opus 4.1 (2025, Anthropic, San Francisco, CA, USA) are some of the most current and advanced models accessible to the wider public. ChatGPT-5 has reportedly improved reasoning and reliability compared with its earlier models, reducing the rate of errors [6,7]. Gemini 2.5 Pro now utilises multimodal data inputs, including text, audio, and visual data, making it particularly applicable in medical diagnostics [8]. Claude Opus 4.1 has a reported focus on safety, precision, and excelling in coding and reasoning [9]. 

Evidence surrounding the applications of using AI in healthcare has been promising amongst multiple medical specialities, including cardiology, radiology, and chronic diseases, with studies showing improvements in patient care, diagnostic accuracy, treatment optimisation, and cost reduction [10-12]. Ophthalmology as a field particularly demonstrates strong potential for the use of AI due to its heavy reliance on image interpretation to reach diagnoses. There is a rising demand for the use of AI in non-ophthalmology settings, including general practice, general medical wards, and emergency departments, where access to urgent ophthalmology advice is variable across locations [13]. Ophthalmology services are predicted to rise in demand, and thus, AI is a tool which could alleviate pressure on healthcare systems if deemed a reliable clinical resource [14,15]. It has already been demonstrated to be useful in the interpretation of fundus photographs, optical coherence tomography (OCT), and fundus fluorescein angiography (FFA) imaging [16-18]. Therefore, there is some evidence in the literature which supports the use of AI in the diagnosis of ophthalmic conditions from images, aiding triage and referral processes. 

However, AI diagnostics based on text-only prompts alone can be more challenging in ophthalmology due to the overlap of signs and symptoms, such as red eye, pain, photophobia, and decreased vision, across several conditions [19]. Correct recognition of ophthalmic emergencies for prompt investigations and management is essential to reduce the risk of permanent complications, including progression to permanent sight loss, and even patient fatality [20-22]. Though some studies show the promising potential of AI in the accurate diagnosis and safe management of medical emergencies, the evidence base is mixed, and its applications within ophthalmology remain a point of uncertainty. Previous research evaluating ChatGPT-4’s performance in the Taiwan Advanced Medical Licensing Examination found that although the model generally performed well, the highest number of mistakes were within ophthalmology [23]. AI models, though continually improving, can often generate inaccurate, misleading information when given medical prompts for interpretation [24]. This highlights the need for further studies assessing whether LLMs can deliver reliable and safe ophthalmic advice to healthcare professionals, particularly in emergency situations, where errors could provide detrimental outcomes for patients. Careful evaluation of the reliability and accuracy of modern AI systems is warranted to identify their safety profile prior to widespread usage of AI within the field of ophthalmology. 

This study aims to evaluate and compare the performance of three LLMs: ChatGPT-5, Google Gemini 2.5 Pro, and Claude Opus 4.1 in the accurate and safe diagnosis, urgency triage, and initial management of ophthalmic emergencies, using standardised text-only clinical vignettes.

## Materials and methods

Clinical case creation

A total of 40 vignettes were created to model common presentations of ophthalmic emergencies (see Appendix 1), evenly distributed across five major subspecialities: cornea and external disease, retina and vitreous, glaucoma and anterior segment, orbit and oculoplastic disorders, and neuro-ophthalmology (Table 1). The choice of 40 vignettes was to ensure representation of a broad range of conditions across each of the subspecialities, whilst also balancing the practical workload of meticulous response scoring. All vignettes were text-only to allow for consistency across the 40 cases. Cases were created using evidence-based clinical sources detailing the common symptoms, signs, aetiology, and examination findings for the ophthalmic emergencies [25-27]. Vignettes were standardised by including details of the patient’s age and sex, a summary of the presenting symptoms, clinically relevant examination findings, and key contextual information for each clinical case. Clinical guidelines for the best-practice, evidence-based management for each vignette were sourced and collated for each of the vignettes to be used as the gold-standard answers, against which the LLM responses will be compared [25-27]. Each clinical case - including the vignettes, most likely diagnosis, urgency status, and management principles - was reviewed and approved by two independent ophthalmologists to ensure accuracy of the cases prior to their usage in the study. Red flags, in this context, have been defined as symptoms, signs, or findings indicating sight-threatening or life-threatening pathology.

**Table 1:** Summary of the 40 conditions represented by the author-generated clinical vignettes used for each of the LLMs LLM: Large language model

Ophthalmology subspeciality	Cases for vignettes
Cornea	Corneal abrasion, Bacterial keratitis, Herpes simplex epithelial keratitis, Bullous keratopathy, Photokeratitis, Corneal foreign body, Keratoconjunctivitis sicca
Retina	Rhegmatogenous retinal detachment, Branch retinal artery occlusion, Central retinal artery occlusion, Vitreous haemorrhage, Wet age-related macular degeneration, Macular hole, Central retinal vein occlusion, Hypertensive retinopathy
Glaucoma	Acute angle-closure glaucoma, Acute anterior uveitis, Acute endophthalmitis, Traumatic hyphaema, Phacomorphic glaucoma, Steroid-induced glaucoma, Congenital glaucoma, Scleritis
Orbit	Orbital cellulitis, Periorbital cellulitis, Globe rupture, Orbital floor fracture, Acute dacryocystitis, Orbital tumour, Cavernous sinus thrombosis, Thyroid eye disease
Neuro-Ophthalmology	Optic neuritis, Anterior retrobulbar optic neuritis, Arteritic anterior ischaemic optic neuropathy, Papilloedema due to raised intracranial pressure, Aneurysmal third-nerve palsy, Weber syndrome, Pituitary apoplexy, Non-arteritic anterior ischaemic optic neuropathy

LLM selection

LLMs were selected based on their global popularity, accessibility, and appropriateness for use in this study context. Only models compatible with the English language were considered. ChatGPT, Claude, and Gemini were identified as the most widely used LLMs and were thus the three models selected. The most advanced available models of each LLM were selected in order to obtain clinically relevant results, which more accurately reflect the modern capabilities of AI; ChatGPT-5, Claude Opus 4.1, and Google Gemini 2.5 Pro were the LLM models selected for the study.

LLM evaluation and response collection

Each vignette was entered into a new conversation within each LLM, using a standardised prompt: ‘You are an ophthalmologist in the United Kingdom. The following patient is presented to you: (VIGNETTE). Answer the following questions only, producing a text-only response: (1) What is the single best diagnosis for this patient? (2) Grade the clinical urgency as either an emergency, urgent, or routine. (3) Outline the key management principles for this patient. (4) Specify whether red flags are present in this case, and provide details if appropriate.’

All LLMs were accessed using Google Chrome (Google, Inc., Mountain View, CA, USA) in one session during September 2025. New conversations within each LLM were used for each vignette, and browser sessions were refreshed between vignette entries to prevent any in-session conditioning, accumulated chat memory, or potential carryover effects from previous cases. Given that LLMs can adapt to multi-turn conversations, a new conversation for each vignette was used to allow for a more objective comparison of the unconditioned decision-making for each of the three models, reflecting baseline model behaviour. All inputs were restricted to plain text; no images, charts, links, or tables were used. Each raw response generated from the LLM was then translated into a spreadsheet for subsequent scoring. 

Response scoring

Each vignette response from each LLM was graded against scoring criteria, devised by the study authors, assessing four domains: diagnosis (0-2 points), urgency classification (0-2 points), management advice (0-3 points), and ability to recognise red flags (0-1 points) (Table 2). A minimum of 0 points and a maximum score of 8 points were available for each LLM response per clinical vignette. Each LLM response was independently scored using the predefined scoring rubric. Where discrepancies in scoring arose, these were resolved through discussion and consensus. The final agreed score, after consensus, was reported and used for all data analysis.

**Table 2:** Scoring criteria for grading LLM responses to clinical vignettes Credit: Created by the study authors Diagnosis was graded on a three-point scale (0-2), urgency on a three-point scale (0-2), management on a four-point scale (0-3), and red flags on a two-point scale (0-1). Minimum total score: 0 and maximum total score: 8. LLM: Large language model

Vignette scoring	Criteria
Diagnosis	
2	Correct or exact likely diagnosis
1	Partially correct or plausible diagnosis
0	Incorrect or dangerously misleading diagnosis
Urgency	
2	Correct classification (emergency, urgent, routine)
1	Partially correct classification
0	Incorrect classification
Management	
3	Entirely correct management advice
2	Mostly correct advice but misses an important step/detail
1	Some correct advice but significantly important advice is omitted
0	Incorrect/harmful advice
Red Flags	
1	Able to identify red flags when present
0	Fails to identify red flags when present

Data analysis

Data was recorded using Microsoft Excel 365 (Microsoft® Corp., Redmond, WA, USA), and statistical analysis was conducted using GraphPad Prism (version 10.6.1; GraphPad Software, San Diego, CA, USA). Descriptive statistics for the quantitative scoring data across LLMs were presented as mean ± standard deviation (SD). The Wilcoxon signed-rank test was used to compare scores for each category and total scores between the three LLMs, with p < 0.05 considered to be statistically significant. Friedman’s test was used to compare overall scores between each LLM for each subspeciality.

Ethics statement

All 40 vignettes used in this study were author-generated, hypothetical scenarios of common ophthalmic emergencies. Any resemblance of vignette content to any patient or any subject is coincidental. No patients or human participants were involved in the creation or assessment of the clinical vignettes or in any other aspect of the study. Two senior ophthalmologists reviewed each of the vignettes solely for content validation and did not contribute any data to the study as participants. In accordance with the Health Research Authority (HRA) UK Policy Framework for Health and Social Care Research, and the UK Research and Innovation (UKRI) guidance, this work therefore did not require formal approval from an ethics committee.

## Results

**Figure 1:**
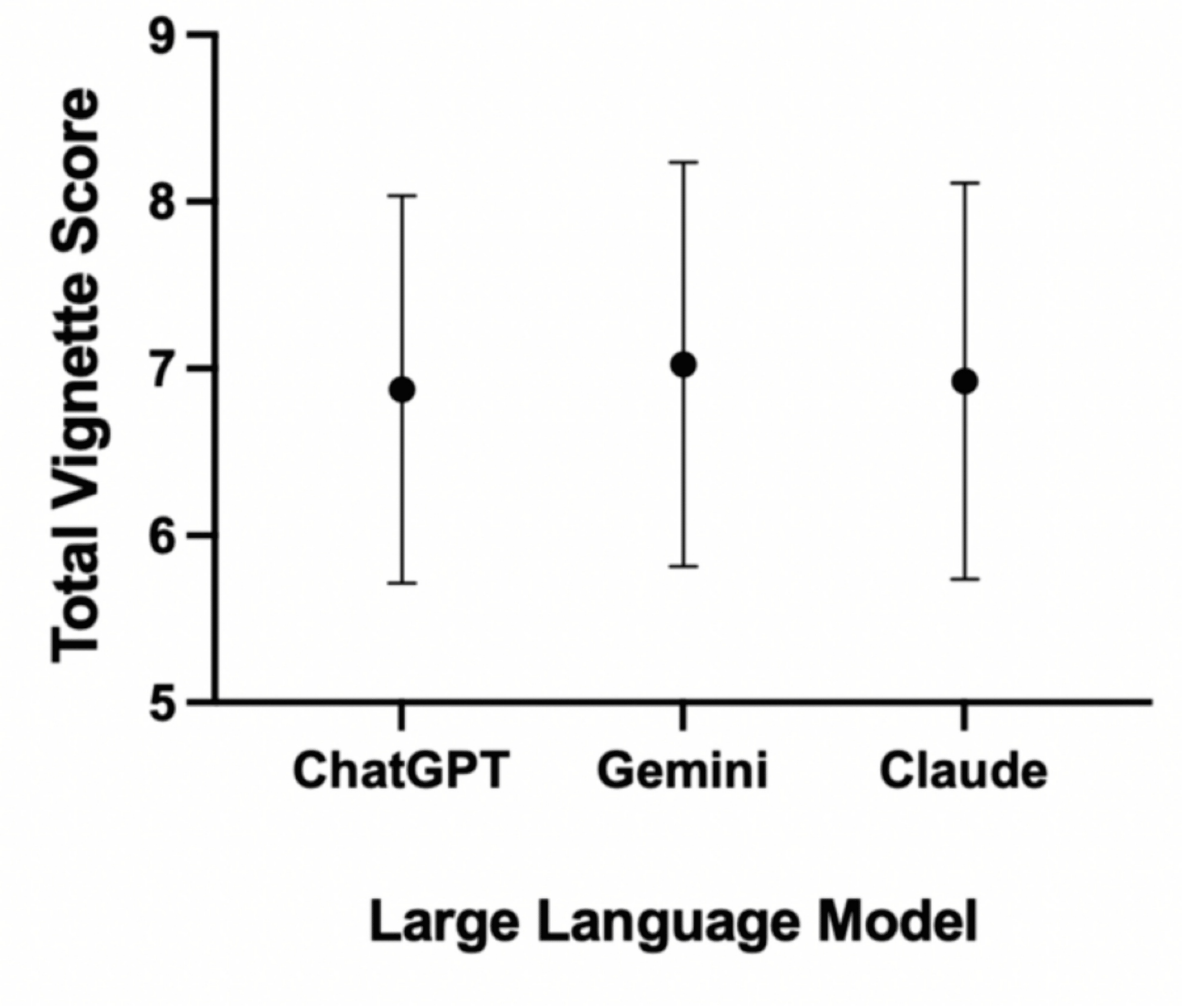
Dot-and-whisker plot demonstrating the mean total scores for the vignettes from ChatGPT-5, Google Gemini 2.5 Pro, and Claude Opus 4.1 Mean values for each LLM are represented by dots, and minimum and maximum standard deviation values are represented by whiskers. LLM: Large language model

A total of 40 clinical case vignettes were inputted and evaluated by ChatGPT-5, Google Gemini 2.5 Pro, and Claude Opus 4.1, totalling 120 responses across all LLMs. Claude achieved the highest absolute diagnostic accuracy of all LLMs, with 35 correct diagnoses out of a possible 40 (87.5%), followed by ChatGPT with 34 (85%), and Gemini with 32 (80%) correct diagnoses (Table 3). Similarly, Claude achieved the greatest mean diagnostic score of 1.88 ± 0.33, compared with 1.85 ± 0.36 for ChatGPT and 1.83 ± 0.38 for Gemini. None of the vignettes across any of the three models received a diagnostic score of 0. Urgency classification scores were consistent across models, with ChatGPT-5 achieving 1.83 ± 0.38, Gemini 1.78 ± 0.48, and Claude 1.75 ± 0.44, with none scoring 0 for any of the vignettes. Gemini was the superior LLM for mean management scores, with a mean of 2.50 ± 0.72, succeeding Claude’s score of 2.35 ± 0.70, and ChatGPT with 2.25 ± 0.81. Red flag recognition was identically high for ChatGPT-5 and Claude (0.95 ± 0.22), but marginally lower for Gemini (0.93 ± 0.27). When combining the total scores across all domains, Gemini achieved a superior mean total score of 7.03 ± 1.21, compared to 6.93 ± 1.19 for Claude and 6.88 ± 1.16 for ChatGPT (Figure 1).

**Table 3:** Descriptive statistics showcasing the performance of each LLM against scoring criteria LLM: Large language model

LLM	Number of correct diagnoses (n, %)	Diagnostic score (mean ± SD)	Urgency classification score (mean ± SD)	Management score (mean ± SD)	Red flag recognition score (mean ± SD)	Overall score (mean ± SD)
ChatGPT-5	34 (85%)	1.85 ± 0.36	1.83 ± 0.38	2.25 ± 0.81	0.95 ± 0.22	6.88 ± 1.16
Gemini Pro 2.5	32 (80%)	1.83 ± 0.38	1.78 ± 0.48	2.50 ± 0.72	0.93 ± 0.27	7.03 ± 1.21
Claude Opus 4.1	35 (87.5%)	1.88 ± 0.33	1.75 ± 0.44	2.35 ± 0.70	0.95 ± 0.22	6.93 ± 1.19

Using the Wilcoxon signed-rank test, pairwise comparisons were performed to help identify any differences between models for each of the scoring domains. The results show that no statistically significant differences were observed between ChatGPT-5, Gemini Pro 2.5, or Claude Opus 4.1 across any of the scoring domains, including diagnostic score, urgency classification, management advice, red flag identification, or overall score (Table 4).

**Table 4:** Paired performance comparisons between scoring domains of ChatGPT and Gemini, ChatGPT and Claude, and Gemini and Claude The Wilcoxon signed-rank test was used to make pairwise comparisons, with p-values reported in the table. A p-value < 0.05 was deemed statistically significant.

	ChatGPT vs Gemini	ChatGPT vs Claude	Gemini vs Claude
Diagnostic score	>0.9999	>0.9999	0.5000
Urgency classification	0.7744	0.3750	>0.9999
Management score	0.1039	0.5743	0.2130
Red flag recognition	>0.9999	>0.9999	>0.9999
Overall score	0.4065	0.6625	0.5555

To further investigate the strengths and weaknesses of each of the LLMs, the errors distributed across each of the scoring domains were summarised (Table 5). Though complete diagnostic errors were not observed, partial errors in the diagnosis were common across all models. Similarly, errors in the urgency classification were predominantly partial rather than complete, with one complete urgency error observed in the Gemini responses. The management domain displayed the highest overall error burden; complete management errors were infrequent, however, all models frequently missed key or partial management steps. Finally, a small number of complete red flag presentations were not recognised across all LLMs.

**Table 5:** Summary table of errors across diagnosis, urgency, management, and red flag recognition domains for the responses produced by ChatGPT, Gemini, and Claude

Error domain	ChatGPT (n)	Gemini (n)	Claude (n)
Diagnosis			
Complete error (score 0)	0	0	0
Partial error (score 1)	6	7	5
Urgency			
Complete error (score 0)	0	1	0
Partial error (score 1)	7	7	10
Management			
Complete error (score 0)	2	1	0
Key missed management (score 1)	3	2	5
Partial missed management (score 2)	18	13	16
Red flags			
Complete omission (score 0)	2	3	2

Subspeciality comparison

Comparison of mean total scores across the ophthalmic subspecialities showed small differences between the models. Gemini and Claude achieved identical mean scores in corneal and external disease, ChatGPT-5 scored the highest mean scores in retina/vitreous and orbit/oculoplastic disorders, whilst Gemini performed best in the glaucoma/anterior-segment and neuro-ophthalmology cases. The Friedman test was then used to assess the difference in performance between the three LLMs. No statistically significant differences were identified within any of the subspecialities, as p-values were > 0.05, indicating similar performance from each LLM between subspecialities (Table 6).

**Table 6:** Comparison of overall LLM performance across subspecialities The Friedman test was used to make comparisons across overall scores of the five subspecialties across the three LLM models. A p-value < 0.05 was considered statistically significant. LLM: Large language model

Subspeciality	Best performing LLM (mean total)	X^2^ (df = 2)	p-value
Cornea and external disease	Gemini/Claude (tie)	1.6875	0.43009
Retina and vitreous	ChatGPT	1.9375	0.37956
Glaucoma and anterior segment	Gemini	1.9375	0.37956
Orbit and oculoplastic disorders	ChatGPT	0.8125	0.66614
Neuro-ophthalmology	Gemini	1.5625	0.45783

## Discussion

The findings of this study provide a comprehensive comparison of three of the leading LLMs - ChatGPT-5, Google Gemini 2.5 Pro, and Claude 4.1 - in the diagnostic, urgency triage, and management capabilities of AI in ophthalmic emergencies, using text-based clinical case vignettes as inputs. Unlike previous studies assessing the diagnostic capabilities of AI LLMs using multimodal inputs such as fundus photographs, OCT scans, or slit-lamp images, this study purely uses text-only inputs to assess how LLMs interpret written clinical information alone [28,29]. To our knowledge, this is one of the first studies that compares these three advanced models in the context of acute ophthalmic presentations using text-based data sources only. Previous work has primarily focused on the assessment of examination-style questions or more narrow ophthalmic contexts, such as the work of Tailor et al., which compared the responses from five older LLMs in the neuro-ophthalmology context for response quality and empathy [30,31]. A recent study in 2025 compared LLMs with the virtual NHS 111 ophthalmology triage pathway, assessing their capability against pre-defined triage algorithms; however, it did not evaluate diagnostic accuracy or management advice in realistic clinical scenarios [32]. Therefore, our findings provide a valuable and updated insight into the current strengths, limitations, and potential clinical applications of LLMs to potentially support a full clinical decision-making pathway in the broader emergency ophthalmology setting. 

Diagnostic accuracy

All three LLMs demonstrated high diagnostic performance, achieving overall accuracies of 80% or more. Claude achieved the highest diagnostic accuracy of 87.5%, followed by ChatGPT-5, scoring 85%, and Gemini, 80%; however, no statistically significant differences were identified between the models on pairwise testing. None of the vignettes across all 120 responses achieved a diagnostic score of 0 points, indicating that all models provided at least a partially correct, reasonable diagnosis for each clinical case. These results suggest that the current generation of LLMs may be able to assist in the identification of many common ophthalmic emergencies from text-based vignettes. However, despite their strong overall diagnostic accuracy, all models occasionally missed key diagnoses, particularly when disease symptoms were subtle or when presentations occurred early in the clinical course of the disease. For example, all three models failed to detect the early signs of bacterial keratitis, in which peripheral corneal infiltrates were not as recognisable as a central corneal opacity as a sign of bacterial keratitis in symptomatic contact-lens wearers. It was noted that the LLMs generally had poorer diagnostic capabilities when common ‘buzzwords’ were omitted from the text prompts, demonstrating the way in which AI uses pattern recognition, as opposed to true cognitive clinical reasoning [33]. This was further reflected by the LLMs demonstrating difficulty in correctly diagnosing less common and more complex clinical conditions, including macular hole and steroid-induced glaucoma, likely due to the reduced information available for the AI models to utilise for pattern recognition.

Furthermore, all models incorrectly assumed idiopathic intracranial hypertension (IIH), as opposed to papilloedema, when an IIH diagnosis cannot be made until other causes of raised intracranial pressure have been appropriately ruled out with imaging and investigations. LLMs are known to occasionally generate fabricated data or use assumed, false knowledge in their clinical reasoning [34]. Aljamaan et al. developed a reference hallucination score (RHS) system, in which ChatGPT scored the highest for hallucination levels across the six chatbots assessed [34]. Until these fictitious outputs are reduced or eliminated from LLM responses, AI cannot be a safe tool in clinical practice without senior-clinician oversight to identify the errors generated.

An example of poorer diagnostic accuracy noted in our study was that both Gemini and Claude confused branch retinal artery occlusion with branch retinal vein occlusion, highlighting a limitation in differentiating two similar clinical presentations based on text alone. The absence of images may also contribute to poorer LLM performance, though this may also reflect typical clinical practice within ophthalmology due to the heavy reliance on imaging and visual assessment in reaching an accurate clinical diagnosis.

These results highlight that, although LLMs show promising diagnostic capabilities, they remain limited in their recognition of subtle contextual details and early disease features that clinicians use when forming a diagnosis. 

Urgency recognition and red flag detection

All three models performed well in the urgency recognition domain, with no vignettes receiving a score of 0. The narrow range of high mean urgency scores, with no statistical significance between any of the LLMs in this category, suggests that all three models were able to prioritise vision-threatening conditions effectively. In practice, this implies that LLMs may have the potential to help support non-specialists in differentiating urgent from non-urgent presentations, helping referral pathways and reducing the time taken for patients to receive ophthalmic care. Current literature similarly supports the use of AI in the urgency triaging of ophthalmology presentations, with Lyons et al., Chen et al., and Ahn demonstrating the high triage accuracy and robust severity classification of AI systems [35-37]. 

Similarly, red-flag recognition was high overall, though a small number of complete omissions were identified. Two vignettes received a red-flag score of 0 for both ChatGPT and Claude, and three for Gemini, highlighting areas of missed recognition of critical features. Although mean red-flag scores remained high, the absent detection of red flags in some clinical presentations potentially devalues the use of AI in automated triage; failure to recognise red flags can lead to devastating, and sometimes fatal, consequences for the patient, particularly in ophthalmology. Missed red-flag signs of raised intracranial pressure can lead to cerebral ischaemia or herniation, and missed red flags for conditions such as optic neuritis can result in irreversible sight loss [27]. 

Management advice

All three LLMs performed well in producing management advice for the clinical vignettes, with mean scores above 2 across all three models. Whilst some variations were observed, with Gemini producing the highest mean management score (2.50 ± 0.72), marginally outperforming Claude (2.35 ± 0.70) and ChatGPT (2.25 ± 0.81), these differences were not statistically significant, suggesting that all three models were capable of providing initial management recommendations for the majority of emergency vignettes. The overall management scores highlight the ability of LLMs to deliver clinically relevant recommendations. With further research and adaptations, LLMs may eventually serve as supportive adjuncts for non-specialist healthcare workers or in settings where specialist advice is limited. This may be particularly applicable in emergency settings, where delays could risk visual outcomes, or in remote environments, where specialist input is not immediately available.

Whilst most responses were thorough, there were some instances in which key omissions in the advice provided could potentially compromise patient safety. In clinical settings, these missed management steps by LLMs would likely be identified by an ophthalmologist or senior clinician. This oversight highlights scope for further research and development in order to make LLM-generated responses at least as safe as initial management plans provided by senior clinicians in the field. Furthermore, to ensure safe application in a clinical setting, LLMs need to be continuously validated against established and updated management guidelines relevant to UK clinical practice to provide reliable and supportive clinical guidance.

Clinical relevance

The consistency across LLMs in our study indicates that all three models can perform with a high level of accuracy and reliability, showcasing the advancements in the development of the most current AI models. Rather than exposing a particular LLM’s weakness, the results have shown that many modern LLMs are capable of producing reliable clinical information and are consistent in their ability to interpret complex clinical text. Their overall performance supports further exploration of LLMs as a supportive adjunct in clinical ophthalmology. Recent studies support this potential, demonstrating that AI systems can improve workflow efficiency, assist in diagnostic decisions, and achieve high accuracy when screening for various ophthalmology diseases [38]. Furthermore, embedding these LLMs into electronic patient record (EPR) systems could, in the future, potentially automatically generate differential diagnoses and management considerations based on clinicians’ notes, subject to clinician review and validation, thus possibly improving the speed and consistency of patient assessment. Such integration could be particularly helpful in emergency departments, where patient turnover is high. 

Moreover, given that each model demonstrated slightly different strengths - with Claude achieving the highest diagnostic accuracy, Gemini performing best in management advice, and ChatGPT excelling in some subspecialities - future integration could benefit from combining the best features of different LLMs. Such an approach could help improve reliability and balance individual model weaknesses, enhancing its performance in clinical applications. In support of this, a systematic review published in April 2025 reported that ensemble modelling approaches, combining outputs from multiple AI systems, have shown improved accuracy and reliability when compared to a single-model framework [39]. 

Beyond direct clinical use, LLMs have an increasingly important role in medical education. The findings of our study support the educational value of LLMs, as they mirror the key elements of clinical reasoning that ophthalmic trainees are expected to develop. In line with this, Antaki et al. reported that ChatGPT has demonstrated performance comparable to that of a first-year ophthalmology doctor on standardised ophthalmology examinations [40]. This shows the potential capability of LLMs in supporting ophthalmic trainees in their diagnostic reasoning and structured decision-making, whilst also recognising that supervised clinical experiences remain paramount to specialist training. 

Limitations of LLMs

Although LLMs show substantial potential in supporting clinical decision-making, their current diagnostic capabilities are limited due to difficulty identifying borderline presentations, which require deeper contextual reasoning beyond simple pattern recognition. Furthermore, although this study did not specifically evaluate reproducibility, it remains a recognised limitation of LLMs. Previous research has shown that identical prompts run under controlled variable conditions can still produce varied outputs [41]. This poses challenges for standardisation and validation, which are crucial in the context of ophthalmology, thus highlighting a barrier to clinical implementation. 

A crucial concern associated with using LLMs in medicine is the issue of liability and accountability, particularly for medicolegal purposes. Using LLMs in healthcare decision-making often raises questions regarding the placement of liability in the event of patient harm, misdiagnosis, or poor clinical management. As outlined by Naik et al., responsibility becomes unclear when AI systems contribute to medical decision-making, as to whether errors should be attributed to the clinician or the AI developer [42]. This uncertainty highlights the need for clear governance frameworks and human oversight to guarantee the safe use of LLMs in clinical settings. 

Limitations of this study

Our study is limited by its non-randomised, non-blinded design, thus introducing a potential for bias in the scoring of the LLM responses against the standardised criteria created by the authors. Blinding of the LLM identity during response scoring, and randomising the order in which the LLM outputs were presented, could have prevented expectation bias or scoring fatigue. 

Secondly, the scoring system used in this study was designed by the authors, as no validated assessment tool exists for evaluating LLM performance in clinical practice. Whilst the designed framework allowed structured and consistent scoring across the different domains, it remains a subjective approach and limits the comparability of results with other studies. Although each of the outputs was evaluated by two reviewers against predefined scoring criteria, there was inevitably an element of subjectivity, where reasoning can vary between the reviewers. Although all discrepancies were subsequently resolved through consensus, as mirrored in routine clinical practice, future studies would benefit from the inclusion of quantitative agreement metrics, such as Cohen’s kappa statistic, to further characterise scoring more objectively.

This study also included only 40 clinical vignettes. Whilst these attempted to cover a range of ophthalmic subspecialities and conditions, they may not fully capture the breadth and variability of clinical ophthalmic presentations in the emergency setting. Furthermore, all 40 vignettes used in this study were created by the study authors. Though efforts were made to minimise bias by involving verification by two independent ophthalmologists, it is important to note the potential for the introduction of significant design bias in this study. Additionally, cases were designed to be moderately stereotypical presentations of the relevant ophthalmic emergency, and, though useful as an introductory assessment of the LLMs in this context, these cases may not reflect the real-world variability seen in emergency eye departments. The inclusion of guideline cues in the phrasing of vignettes, such as examination signs unique to a particular ophthalmic condition, may have influenced the model accuracy demonstrated in the results of this study.

This study was also limited to text-based clinical vignettes and, therefore, did not assess the multimodal abilities of these LLMs. Ophthalmology is a field that relies heavily on visual assessment, such as slit-lamp findings, fundus images, and OCT scans; excluding these elements from the vignettes limits their real-world applications and restricts the LLMs from assessing imaging-dependent conditions. Hence, our findings reflect performance in text-only diagnostic reasoning rather than the full spectrum of ophthalmic assessment encountered in clinical practice.

When inputting prompts into each of the LLMs, the same standardised prompt was used across all vignettes, and each vignette was used only once within each LLM in a new conversation without any prior conversational warm-up. Though this approach reflects real-world utility in first-use scenarios, it does not assess the reproducibility of the results, nor does it evaluate the effects of prompt conditioning with prior conversational warm-up. There is potential for LLMs to produce variable output responses across repeated runs with identical prompts - a factor which was not assessed in this study. Future studies should consider using repeated runs of the vignettes to better assess reproducibility, consider the use of conversational warm-up to assess if different responses are produced, and investigate the effect of using alternative prompt structures for the same vignettes to assess output stability.

Finally, given that LLMs are continuously improving and developing, the findings of this study are time-sensitive. The performance observed in this study may not be reproducible in future versions. As newer and more advanced models continue to be developed, re-evaluation may be required to determine whether diagnostic accuracy, reasoning ability, and clinical application continue to improve. 

Strengths of this study

At the time of writing, to our knowledge, this is one of the first studies comparing these three leading LLMs - ChatGPT, Google Gemini, and Claude - in the diagnosis and management of emergencies in ophthalmology using text-only case vignettes. Though studies evaluating the usage of novel prototype AI models in the ophthalmic emergency context were identified, we were unable to find research papers comparing the existing leading LLMs for the evaluation of text-based vignettes within ophthalmic emergencies [43]. Our research adds valuable evidence to the field, highlighting the equity of ChatGPT, Google Gemini, and Claude in their applications to ophthalmology decision-making, particularly in the emergency context. We provide evidence that all three AI models are capable of providing suggestions for diagnosing, triaging urgency, and providing management advice, which may assist clinicians as an adjunct in their clinical practice. Overall scores are high across all LLMs, and, with no statistical differences identified across scoring categories or between subspecialities, we are able to add supportive evidence to the field for using any of the three leading LLMs in the ophthalmology context.

Furthermore, our study utilises the most advanced models of each of the three LLMs at the time of writing, showcasing the maximum capabilities of modern AI in this context and producing the most meaningful results possible to date. Though AI models continue to develop, we produce results representing the greatest capabilities of AI currently due to the selection of these advanced models. This aspect of our study design ensures our results will remain clinically valid for longer than studies using outdated, free models.

Utilising 40 of the most common ophthalmic emergencies of varying difficulties, and across five widely categorised subspecialities, demonstrates the useful application of our findings across the general field of ophthalmology. Clinical cases were created using the most updated, evidence-based guidelines relevant to UK clinical practice, thus assessing the capabilities of AI LLMs to produce the most relevant management suggestions for each of the suspected diagnoses. The inclusion of independent ophthalmologists in the review process strengthens the internal validity of our findings and ensures that the clinical cases, and results produced from using the cases, reflect accurate clinical presentations of ophthalmic emergencies and evidence-based management plans.

Finally, the quantitative scoring assessment utilised in this study allows for objective assessment and comparison across each of the AI models and subspeciality domains. This objective scoring allows for fair comparison between the LLMs and reduces the likelihood of bias being introduced, compared to the use of more subjective, qualitative evaluation tools. 

Future research scope

The promising results of our study justify further research into the use of different LLMs in supporting clinicians with the diagnosis and management of clinical presentations within ophthalmology. Future clinical work should focus on the comparison of text-based vignettes in combination with multiple forms of multimodal media, such as Humphrey’s visual fields, OCT images, fundus photographs, and FFA imaging. This would more accurately emulate the real-world clinical environment in which an ophthalmologist would be making similar clinical decisions, and would thus test the AI as a truer comparison to decision-making at the level of an ophthalmologist. 

Clinical scenarios should be expanded to incorporate an even broader range of clinical cases of varying complexity and clinical urgency, to further assess the ability of AI in the recognition of less common conditions and atypical presentations of common conditions. A wider range of cases will better simulate real-world diagnostic uncertainty and variation in clinical practice. Moving beyond author-generated vignettes to the assessment of anonymised patient encounters may yield more applicable results, with greater clinical relevance, and provide a greater measure of utility and safety of LLMs in real clinical practice.

Additional work in the field should, importantly, progress beyond simulated scenarios and investigate AI in real ophthalmology settings, using authentic clinical data, referral letters, and information from the wider EPRs. Examining the use of AI in live clinical workflows would provide important insights into their ability to be integrated into real-world clinical encounters. Their use in different settings, such as primary care and community optometry, will help obtain a more practical understanding of the potential of LLM systems and establish true clinical utility and safety in decision-making within ophthalmology.

## Conclusions

This study provides direct comparisons between three advanced LLMs - ChatGPT-5, Google Gemini 2.5 Pro, and Claude Opus 4.1 - in the diagnosis, urgency triage, and initial management of ophthalmic emergencies using text-based clinical vignettes. All three LLMs demonstrated consistent accuracy across all evaluated domains, with no statistically significant differences in overall performance. These findings highlight the rapid progress of current LLMs in interpreting complex ophthalmic presentations and delivering preliminary diagnostic and management suggestions, which may support clinical decision-making in the emergency context. By supporting early recognition of urgent conditions and guiding initial management, these models have the potential to aid ophthalmic triage and clinical decision-making, particularly for non-specialist settings with limited ophthalmology expertise available at short notice.

However, as there were diagnostic inaccuracies and missed management details, continued refinement, validation, and evaluation within clinical practice remain essential, and highlight that LLMs should only be used as a clinical adjunct, not in isolation. Overall, our findings add further evidence supporting the incorporation of LLMs in modern clinical practice as supportive tools in the delivery of ophthalmic emergency care.

## Appendices

Appendix 1

**Table 7:** Full list of 40 vignettes, with subspecialty, diagnosis, clinical urgency and key management advice Created utilising guidelines aligned to UK clinical practice, as detailed in the Methods section.

Vignette number	Vignette	Speciality	Diagnosis	Clinical urgency	Key management
1	A 28-year-old man presents with sudden grittiness and pain in the left eye with excessive tearing after scratching his eye with his fingernail.	Cornea	Acute corneal abrasion	Urgent	Removal of foreign body, if present; topical antibiotics (e.g., erythromycin ointment, ciprofloxacin drops, or ofloxacin drops); oral analgesia; consider topical cycloplegic (e.g., atropine)
2	A 30-year-old contact lens-wearer reports a mildly uncomfortable and red left eye, with mild peripheral corneal infiltrates seen on slit-lamp.	Cornea	Bacterial keratitis (early)	Emergency	Topical antibiotics (e.g., gentamicin + vancomycin, or moxifloxacin, or levofloxacin); discontinue contact lens wear during therapy; consider cycloplegic eyedrops for symptom relief (e.g., atropine); consider offering oral analgesia
3	A 24-year-old contact lens-wearer presents with an acutely red and painful eye and mucopurulent discharge, with a central corneal opacity and corneal thinning visualised on examination.	Cornea	Bacterial keratitis (Pseudomonas)	Emergency	Topical antibiotics to cover Pseudomonas infection (e.g., gentamicin + vancomycin, or moxifloxacin, or levofloxacin); discontinue contact lens wear during therapy; eye shield due to the presence of corneal thinning; consider cycloplegic eyedrops for symptom relief (e.g., atropine); consider offering oral analgesia
4	A 37-year-old presents with a painful, watery left eye and inflamed eyelid margins, with a branching corneal ulcer visualised on fluorescein staining.	Cornea	Herpes simplex keratitis	Urgent	Topical antiviral therapy (e.g., ganciclovir gel, aciclovir ointment); oral aciclovir if unable to tolerate topical; consider anti-glaucoma therapy if intraocular pressure (IOP) is raised; consider topical polymyxin B/trimethoprim; consider cycloplegic eyedrops for symptom relief (e.g., atropine); consider offering oral analgesia
5	An 88-year-old presents with gradual worsening of vision in his pseudophakic right eye, worst in the morning, with some corneal oedema visualised on slit-lamp.	Cornea	Bullous keratopathy	Routine	Hypertonic eye drops and ointment (e.g., 2–5% sodium chloride) with increased frequency in the mornings; bandage contact lens for comfort; consider lubricant eye drops/ointments for ruptured epithelial bullae; consider topical antibiotics for ruptured epithelial bullae; corneal transplantation surgery as definitive treatment; offer oral analgesia
6	A 37-year-old welder presents with bilateral eye pain, photophobia and eyelid erythema 2 hours after his shift ended.	Cornea	Photokeratitis	Urgent	Supportive treatment - should resolve within 72 hours; offer topical ointment, artificial tears, and oral analgesia to treat symptoms; consider topical antibiotics, e.g., erythromycin ointment, for comfort and prophylaxis; advise future UV protection to avoid recurrence
7	A 45-year-old builder presented with sudden-onset eye pain, watering and irritation whilst working outside.	Cornea	Corneal foreign body	Urgent	Prompt removal of foreign body from the surface; topical broad-spectrum antibiotics, e.g., chloramphenicol ointment; consider bandage contact lens for comfort; consider cycloplegic eyedrops for symptom relief, e.g., atropine; offer advice to prevent future injuries, including wearing safety glasses
8	A 19-year-old with previous bilateral laser-assisted in situ keratomileusis (LASIK) surgery presents with itchy, gritty and red eyes, which are often sensitive to light.	Cornea	Keratoconjunctivitis sicca	Routine	Topical lubricants and artificial tear eyedrops; eye ointments at night; severe cases - consider topical corticosteroids; refractory to topical therapy - consider lacrimal punctal plugs; consider moisture chamber spectacles
9	A 76-year-old with a history of diabetes and hypertension presents due to a sudden-onset change in his vision, described as a veil coming across his eyes with some flashes of light.	Retina	Rhegmatogenous retinal detachment	Emergency	Urgent surgery under the ophthalmology (vitreoretinal) team: scleral buckle and/or pars plana vitrectomy, or pneumatic retinopexy
10	A 93-year-old patient presents with a painless, sudden drop in vision in her right eye with a 1-line decrease in her Snellen VA. Cotton wool spots are visualised on fundoscopy.	Retina	Branch retinal artery occlusion	Emergency	Urgent referral to a stroke centre for systemic stroke workup and consideration of carotid ultrasound and/or echocardiography; control of risk factors, e.g., hypercholesterolaemia; branch retinal artery occlusion (BRAO) often resolves spontaneously.
11	An 87-year-old man with diabetes, hypertension and obesity presents with more than 2 hours of sudden-onset loss of vision in the left eye. Fundoscopy is normal.	Retina	Central retinal artery occlusion	Emergency	Urgent referral to stroke centre for systemic evaluation for stroke/cerebrovascular accident; consider ocular massage to dislodge embolus; consider lowering intraocular pressure using eyedrops or anterior chamber paracentesis
12	A 43-year-old with poorly controlled type 2 diabetes, atrial fibrillation, giant cell arteritis and psoriatic arthritis describes a sudden-onset drop in vision in his right eye, with a red-tinted hue. The retina cannot be visualised on fundoscopy.	Retina	Vitreous haemorrhage secondary to proliferative diabetic retinopathy	Urgent	Urgent ophthalmology surgery - pars plana vitrectomy as the retina cannot be adequately visualised and the aetiology of the vitreous haemorrhage is unknown; consider intra-operative panretinal photocoagulation or intravitreal anti-VEGF injection for diabetic retinopathy; improve diabetic control
13	A 79-year-old smoker presents with sudden blurred central vision and noticeable distortion of words and lines.	Retina	Exudative (wet) age-related macular degeneration	Urgent	Intravitreal vascular endothelial growth factor inhibitor; consider antioxidant and mineral supplementation; ophthalmologist opinion regarding thermal laser photocoagulation as only used for small extrafoveal choroidal neovascularisation; can consider photodynamic therapy using verteporfin in those with subfoveal choroidal neovascularisation
14	A 62-year-old woman presents with distorted central vision and central vision loss. A dark red spot in the central macula, and intraretinal cystic changes are observed on fundoscopy.	Retina	Macular hole	Emergency	Urgent surgery under the ophthalmology team - pars plana vitrectomy with tamponade
15	An 83-year-old man presents with sudden, severe blurring of vision in one eye, with a marked relative afferent pupillary defect (RAPD) and widespread haemorrhages seen on fundoscopy.	Retina	Ischaemic central retinal vein occlusion	Urgent	Close monitoring plus management of underlying risk factors; if with macular oedema: intravitreal injection of vascular endothelial growth factor inhibitors (e.g. ranibizumab, aflibercept or bevacizumab); if with neovascularisation: pan-retinal photocoagulation
16	A 67-year-old with a known phaeochromocytoma has slowly declining visual acuity, and cotton wool spots are seen on fundoscopy.	Retina	Hypertensive retinopathy	Routine	Improved control of hypertension with oral anti-hypertensive agents; if hypertensive crisis, to be referred to the emergency department for acute antihypertensive management; consider informing the endocrinology team due to symptomatic phaeochromocytoma
17	A 57-year-old man presents with a severe, acutely painful and red left eye, with a mid-dilated pupil and raised intraocular pressure.	Glaucoma/anterior segment	Acute angle-closure glaucoma	Emergency	Topical carbonic anhydrase inhibitors in combination with topical beta blockers and topical alpha-2 agonists; consider topical ophthalmic cholinergic agonists (e.g., pilocarpine); consider hyperosmotic agents if IOP remains over 50 mmHg (e.g., mannitol); laser peripheral iridotomy after acute attack resolved
18	A 42-year-old woman with ankylosing spondylitis and rheumatoid arthritis has severe, aching pain in her right eye, with redness and photophobia. The pupil is small and irregular, and the eye surface redness does not blanch with phenylephrine drops.	Glaucoma/anterior segment	Acute anterior uveitis	Emergency	Topical ophthalmic corticosteroids (e.g., prednisolone, dexamethasone, fluorometholone, difluprenate); consider topical cycloplegics (e.g., atropine)
19	A 77-year-old man who had a routine phacoemulsification 4 days ago presents with severe pain and redness of the eye with visible white cells in the anterior chamber.	Glaucoma/anterior segment	Acute endophthalmitis (postoperative)	Emergency	Urgent admission under ophthalmology for systemic antibiotics and monitoring; anterior chamber fluid tap and intravitreal injection of antibiotics (e.g. vancomycin, ceftazidime); consider pars plana vitrectomy (PPV)
20	A 16-year-old boy presents following a left eye injury whilst playing football at school. He complains of decreased vision in the eye, pain, and photophobia, and red cells are visible in the anterior chamber.	Glaucoma/anterior segment	Traumatic hyphaema	Emergency	Conservative management with an eye shield, limited activity and head elevation; consider topical corticosteroids to reduce associated inflammation; consider topical cycloplegics (e.g., atropine) for ciliary spasm or photophobia; topical aqueous suppressants if IOP is raised; consider systemic agents if topical management fails to control pressure
21	An 84-year-old woman on a delayed waiting list for her cataract surgery presents with an acutely red and painful eye. Visualisation of the posterior chamber is hindered due to the density of the cataract.	Glaucoma/Anterior Segment	Phacomorphic glaucoma	Emergency	Lower IOP with topical medical therapies including topical beta blockers and carbonic anhydrase inhibitors; consider laser iridotomy if pressure is not controlled with topical therapy alone; definitive treatment with surgical cataract extraction (phacoemulsification)
22	A 63-year-old woman with type 1 diabetes, rheumatoid arthritis, and Cushing’s syndrome presents with a slowly worsening, painful, red right eye with raised intraocular pressures.	Glaucoma/Anterior Segment	Steroid-induced glaucoma	Urgent	Discontinue steroid therapy gradually; topical glaucoma medications to lower IOP; consider procedures such as selective laser trabeculoplasty, goniotomy, and glaucoma drainage implant surgery to lower IOP and prevent future IOP spikes
23	A 4-month-old child is brought in due to recurrent episodes of blepharospasm and excessive watering of the eyes. Corneal oedema is noted on anterior examination.	Glaucoma/Anterior Segment	Primary congenital glaucoma	Emergency	Angle surgery (goniotomy or trabeculotomy) to lower IOP by improving aqueous outflow; if angle surgery is not successful, consider trabeculectomy enhanced with mitomycin C or glaucoma implant surgery; medical therapy (topical or oral) to lower IOP whilst awaiting surgery
24	A 42-year-old woman with ankylosing spondylitis and rheumatoid arthritis has severe, aching pain in her right eye radiating to her forehead, with photophobia and tender scleral redness, which does not blanch with phenylephrine drops.	Glaucoma/Anterior Segment	Scleritis	Emergency	Oral non-steroidal anti-inflammatory drugs (NSAIDs) (e.g., ibuprofen, indomethacin) with oral proton pump inhibitor (PPI) cover; consider systemic corticosteroids to further decrease ocular inflammation; immunomodulatory agents (e.g., methotrexate, mycophenolate, or cyclophosphamide) if disease remains inadequately controlled on corticosteroids
25	A 14-year-old boy is unwell with a fever, nasal sinus infection, and a painful swelling around the left eye, with painful eye movements and photophobia.	Orbit/Oculoplastics	Orbital cellulitis	Emergency	Hospitalisation for empirical or targeted intravenous antibiotics (dependent on whether the causative organism identified), e.g., cefotaxime, clindamycin, cefuroxime, metronidazole therapy; consider nasal decongestant; consider systemic corticosteroids; consider empirical antifungal therapy in immunosuppressed or ketoacidotic patients; consider lateral canthotomy and cantholysis if high IOP; consider orbitotomy and surgical drainage of orbital abscess
26	A 3-year-old boy presents with mild right eyelid swelling. He is clinically otherwise well and has normal cranial nerve examination findings.	Orbit/Oculoplastics	Periorbital (preseptal) cellulitis	Urgent	Empirical/targeted oral or intravenous antibiotic therapy (dependent on if causative organism identified); consider incision, drainage, and culture of peri-ocular abscess; consider empirical antifungal therapy
27	A 34-year-old man suffers an injury to his left eye during a hockey match. On examination, he is in significant pain and has widely dispersed subconjunctival haemorrhages and an irregular pupil.	Orbit/Oculoplastics	Globe rupture	Emergency	Immediate surgery under the ophthalmology team - ruptured globe repair; preoperative protection against external pressure with a shield; systemic anti-emetics to reduce risk of raised IOP; prophylactic antibiotics to cover for post-traumatic endophthalmitis (e.g., vancomycin and ceftazidime); topical antibiotics, corticosteroids and cycloplegics to treat traumatic uveitis associated with the injury
28	A 65-year-old man who was punched in his left eye presents with diplopia, restricted upgaze, and infraorbital numbness.	Orbit/Oculoplastics	Orbital floor fracture with inferior rectus entrapment	Emergency	Advanced trauma life support (ATLS) and urgent surgical intervention under ophthalmology and maxillofacial/ENT teams; consider 1 week of prophylactic antibiotics if open fracture / surgical emphysema / orbital grafting / open reduction and internal fixation is performed
29	A 42-year-old woman presents with a 3-day history of excessive tearing and discharge production from the right eye, with inferomedial orbital pain.	Orbit/Oculoplastics	Acute dacryocystitis	Urgent	Oral empirical antibiotics with gram-positive coverage, with escalation to IV antibiotics if progression/complicated/orbital cellulitis; address underlying cause with surgical intervention, e.g., dacryocystorhinostomy (DCR) / stenting / balloon dacryoplasty once acute inflammation/infection has resolved
30	A 79-year-old is referred with a painless, slowly worsening proptosis of the left eye and new diplopia.	Orbit/Oculoplastics	Orbital tumour	Urgent	Urgent 2-week-wait referral for suspected orbital malignancy, to obtain neuroimaging and specialist assessment; urgent review by neuro-ophthalmology +/- neurosurgery +/- orbital oncology team for plan regarding observation / radiotherapy / surgical management
31	A 72-year-old man on antibiotics for a dental infection presents with rapidly progressive periorbital swelling, chemosis, and unilateral headache.	Orbit/Oculoplastics	Cavernous sinus thrombosis	Emergency	High-dose IV antibiotics are administered for an extended period of time (3-4 weeks); if no haemorrhagic complications, then consider anticoagulation; surgical drainage of the dental abscess
32	A 43-year-old woman with a history of autoimmune disease presents with protruding eyes, double vision, and grittiness.	Orbit/Oculoplastics	Thyroid eye disease	Urgent	Improve thyroid medical therapy to reach euthyroid status; for corneal exposure: lubricants, taping, and/or protective shields; for diplopia: prisms or occlusion therapy; consider systemic steroids (e.g., oral prednisolone) to decrease orbital inflammation; consider orbital decompression surgery
33	A 24-year-old woman has an acute, painful loss of central vision and decreased colour perception.	Neuro-Ophthalmology	Optic neuritis	Urgent	High-dose intravenous corticosteroids e.g., methylprednisolone, should be initiated within the first 48 hours of onset of symptoms; follow by a tapering dose of oral steroids (e.g., prednisolone); gastric protection should be provided with steroids
34	An 18-year-old presents with acute vision loss in her right eye with mildly painful eye movements, mild colour desaturation in the eye, and normal fundoscopy examination.	Neuro-Ophthalmology	Acute retrobulbar optic neuritis	Urgent	High-dose intravenous corticosteroids, e.g., methylprednisolone, should be initiated within the first 48 hours of onset of symptoms; follow by tapering dose of oral steroids (e.g., prednisolone); gastric protection should be provided with steroids
35	A 56-year-old man presents with sudden-onset vision loss, decreased visual acuity, and RAPD with severe pain in his head and jaw. Fundoscopy shows a swollen, pale optic disc.	Neuro-Ophthalmology	Arteritic Anterior Ischaemic Optic Neuropathy (AAION)	Emergency	Urgent systemic steroids, e.g., IV methylprednisolone for 3 days followed by tapering oral steroids; discussion with both rheumatology and ophthalmology teams for likely Giant Cell Arteritis
36	A 37-year-old woman presents with intermittent headaches, worst in the morning and when bending forward. Her vision is normal and fundoscopy shows bilateral optic disc swelling.	Neuro-Ophthalmology	Papilloedema - disc swelling due to raised intracranial pressure (ICP)	Emergency	Urgent referral to neurosurgery for CT imaging to exclude space-occupying lesion & cerebral venous thrombosis; urgent LP to confirm raised ICP; treat underlying cause of raised ICP if found + lower ICP (e.g., acetazolamide) + offer analgesia; if no cause identified -> idiopathic intracranial hypertension (IIH); manage with weight loss advice if appropriate, avoid precipitating factors, lower ICP (e.g., acetazolamide or topiramate), and offer analgesia
37	A 72-year-old man presents with an acutely painful diplopia, unilateral ptosis, and dilated, minimally reactive pupil, with the eye in an inferolateral position at rest.	Neuro-Ophthalmology	Aneurysmal third-nerve palsy	Emergency	Immediate referral to neurosurgical team due to suspected aneurysm; urgent neuroimaging (e.g., CTA head or MRA head) to exclude compressive cerebral aneurysm; neurosurgical team to assess need for urgent surgical management (e.g., microsurgical clipping or endovascular techniques), or for observation; offer analgesia
38	A 75-year-old ex-smoker with a history of hypertension and cardiovascular disease presents with an acute ptosis and inferno lateral deviation of his right eye, with weakness of his left limbs. Fundoscopy and slit lamp examination are normal.	Neuro-Ophthalmology	Weber syndrome (midbrain infarct resulting in CN III palsy and contralateral hemiparesis)	Emergency	Stabilisation and immediate referral to an acute/hyperacute stroke unit; urgent neuroimaging (CT head) to rule out haemorrhage and confirm infarct/ischaemic stroke; if ischaemic stroke is ruled out, no contraindications and within 4.5h of symptoms: thrombolysis (alteplase or tenecteplase); antiplatelet agents (e.g. aspirin) and statins (e.g. atorvastatin); consider mechanical venous thromboembolism (VTE) prophylaxis and early mobilisation
39	A 60-year-old woman with hypertension and atrial fibrillation develops a sudden-onset severe headache, nausea, and bitemporal hemianopia, before losing consciousness. Fundoscopy examination is normal.	Neuro-Ophthalmology	Pituitary apoplexy	Emergency	Surgical emergency requiring immediate surgical decompression of the sella under neurosurgery team; immediate systemic corticosteroids as soon as pituitary apoplexy is suspected
40	A 76-year-old man with hypertension, sleep apnoea, and chronic obstructive pulmonary disease (COPD) wakes up with sudden, painless unilateral vision loss in his right eye. Fundoscopy shows a swollen optic nerve with peripapillary splinter haemorrhages.	Neuro-Ophthalmology	Non-arteritic anterior ischaemic optic neuropathy (NAION)	Emergency	No effective treatment options available for NAION; consider anticoagulation if thrombosis suspected as cause of presentation; consider systemic corticosteroids (e.g., oral prednisolone) for treatment of disc oedema if suspected as cause of presentation
